# The Effects of Very-Low-Calorie Diets on HDL: A Review

**DOI:** 10.1155/2011/306278

**Published:** 2010-12-22

**Authors:** Catherine Rolland, Iain Broom

**Affiliations:** Centre for Obesity Research and Epidemiology, Robert Gordon University, St. Andrew Street, Aberdeen AB24 3LR, UK

## Abstract

This paper investigates the effects of very-low-calorie diets (VLCDs) used in the treatment of obesity on high-density lipoprotein (HDL) levels. Although the studies varied widely in their intervention format, duration, and baseline HDL levels, it would appear that HDL levels usually decrease during active weight loss using a VLCD, but these either return to pre-VLCD levels or improve overall during the weight-maintenance phase. More research needs to be done to determine optimal weight-maintenance programmes and the effects of VLCDs in the short term as well as on HDL levels in groups at increased risk of coronary heart disease.

## 1. Introduction

With the continuous rise in obesity, there has been resurgence in the use of very low calorie diets (VLCDs) and more research investigating the effects of such diets has been undertaken. VLCDs have often been criticised for being unsafe and an unhealthy way to lose weight. The current VLCDs, however, are not to be confused with those from the 1970s which resulted in a number of deaths, probably due to vitamin and mineral deficiencies and poor quality or inadequate amounts of protein [1, 2]. VLCDs are defined as diet replacements with <800 kcal and >400 to 450 kcal/d [3]. There are now a number of commercially available VLCDs.

Evidence from a number of reviews [3–5] clearly demonstrates that VLCDs can result in significant weight loss. Despite this, there are still concerns about weight regain following these diets as well as detrimental health effects due to the rapid weight loss they induce. In this paper, we aim to examine the literature and to investigate the effects of very low calorie diets on high density lipoproteins (HDL) levels specifically.

## 2. Importance of HDL

Obesity is commonly associated with elevated triacylglycerols, low HDL, and normal low-density lipoproteins [6]. The low HDL observed in obesity is associated with an enhanced risk of atherogenesis possibly due to increased degradation and/or decreased production of HDL particles. Adipose cells have been shown to bind to HDL [7], hence increased body fat may lead to an increased uptake of HDL particles from circulation resulting in a reduction in plasma HDL levels [8]. On the other hand, an increase in HDL has been associated with decreased risk of coronary artery disease [9], probably due to its role in the reverse cholesterol transport process where cholesterol in peripheral tissues is transported to the liver for reuse or bile acid synthesis, preventing the accumulation of cholesterol in the arteries [10]. HDL is also thought to be cardioprotective due to its antioxidant activity [11].

## 3. Effect of VLCD on HDL Levels

Several studies have investigated changes in HDL after a period of VLCD. In Table 1, we report the findings from 5 studies in which participants underwent VLCDs for as little as 2 days and as long as 12 weeks. The calorie intake varied from 450–800 kcal/day. One study [12] looked at the differences in ethnicity in response to a VLCD delivered with exercise and behavioural therapy. Also, the gender composition of the studies were quite different, as some studies looked at women only [12, 13], while the other studies all included men [14–16]. Weight loss was significant in all but the 2-day VLCD group [13].

With the exception of the group that underwent a VLCD for 2 days [13], changes in HDL are reported in Table 1. HDL was significantly decreased in two of the studies [12, 14]. Hong et al. [12] investigated the differences in response between black and white individuals. Their findings demonstrated that there was no difference between these two ethnic groups for weight and HDL in response to the VLCD intervention (which also included behaviour therapy and exercise). 

Two other studies also displayed a trend for a decrease in HDL [13, 16]. In the study by Lin et al. [16], all patients underwent two weeks of an LCD followed by 12 weeks of VLCD. The two groups differed in the caloric intake during the VLCD period (450 versus 800 kcal/day). Weight loss and changes in HDL were not significant between the two groups. 

Only one study reported an increase in HDL after a 9-week VLCD [15], but this was not significant.

## 4. Effect of VLCD and a Weight-Maintenance Period on HDL Levels

A number of studies investigated changes in HDL after a VLCD and a weight-maintenance period (Table 2). These studies varied in duration of VLCD (6 weeks–9 months), maintenance period/followup (4 weeks–5 years). Energy intake for the VLCD varied from 400–800 kcal/d. Two studies included exercise [17, 18], while three other studies included behaviour therapy [19–21], four studies included medication [22–25] and two studies investigated individuals with type 2 diabetes [21, 26]. Weight loss was significant for all studies after the VLCD, although one study [18] included an LCD phase before the VLCD, another study [21] included a refeeding phase before the measurements were taken, and a third study reported BMI but not weight, and BMI change was not reported after the period of VLCD [26]. Weight loss at the end of the study remained significant in 9 of the 12 studies. As mentioned previously, the study by Paisey et al. [26] only reported BMI which did not change significantly. The changes in the study by Fogelholm et al. [18] were more difficult to report for both weight and HDL as the baseline values were reported for all of the participants and not provided separately for each treatment group. In addition, this study did not give within group significance for changes in weight or HDL at followup. Finally, Apfelbaum et al. [25] only report weight change from the time of randomisation after the VLCD to the end of the study, therefore not including the weight loss achieved using the VLCD.

At the end of the VLCD phase, HDL was reported for 9 of the 12 studies. HDL was decreased in at least one of the treatments for 6 of the studies [18, 20, 22, 23, 27, 28]. This was significant in three studies [20, 22, 28] and not reported in two others [18, 23]. Four studies [19, 21, 24, 27] reported an increase in HDL after the VLCD, only one of which was not significant [27], however, the 2 studies that report an increase in HDL had a period of refeeding before the measurement [19, 21]. All of the studies reported an increase in HDL at the end of the study with the exception of Haugaard et al. [28] and one arm in the study by Christiansen et al. [17] which both showed no change in HDL. The increase in HDL was significant in at least one arm of 8 [17, 19–22, 24, 25, 27] out of the 12 studies.

The study by Christiansen et al. [17] reports that exercise results in a significant improvement on HDL while the study by Fogelholm et al. [18] shows a similar trend, but the results were not significant at the 2 year followup.

Delbridge et al. [27], do not report a significant difference in the improvement in HDL at the time of followup between the high-carbohydrate and the high-protein weight-maintenance group. Also, there were no significant differences in HDL between the use of a placebo and Orlistat [22] or between placebo and the neuropeptide 5 receptor antagonist MK-0557 [23] for weight maintenance, but there was a significant improvement in HDL when Sibutramine was used compared to placebo [25].

## 5. Effect of Intermittent Use of VLCD on HDL Levels

Five studies reported the effects of intermittent use of VLCDs on HDL (Table 3). The duration of the studies varied from 14 weeks to over two years. The energy intake from VLCDs ranged from 400–600 kcal/d. Two studies included women only [29] and one study included type 2 diabetic patients only [30]. The format of the different interventions varied widely, and is described in more detail in Table 3. 

Only three studies reported weight loss after the first phase of VLCD [8, 21, 31] and all three studies report a significant weight loss. Weight loss at the end of the study was significant for all five studies.

HDL change was reported after the first phase of VLCD in all 5 studies. At this stage, HDL had decreased significantly in 4 studies [8, 29, 30], and increased in one study [21]. At the end of the studies, HDL was increased in at least one arm of 3 studies [21, 30, 31], only two of which were significant [21, 31]. HDL decreased in at least one arm of 3 studies [8, 29, 30] none of which were significant.

## 6. Discussion

Here we reviewed the limited information available about the effects of VLCDs available. The results suggest that although an improvement in HDL levels during active weight loss using a VLCD is not always present, there often is an improvement observed during weight maintenance. 

It was interesting to see in the study by Lin et al. [16] that there was no significant difference in weight loss and HDL between the group that consumed 450 kcal/d compared to the group that consumed 800 kcal/d. This is consistent with other studies that compared the use of diets ranging from 240–880 kcal/d and found no significant difference in weight loss for short (4–6weeks) and intermediate term (16–26 weeks) [32–34]. In light of this, it could be beneficial for patients to be allowed to consume more and minimise the sense of limitation that occurs when using a VLCD, and use a combination of liquid diet supplemented with lean meats or low-starch vegetable.

From the studies presented in this paper, it appears that HDL initially decreases during weight loss treatment using VLCDs, but then either increases back to baseline levels or result in an overall improvement of HDL levels during weight maintenance. This biphasic response may explain the discrepancies between the studies presented here. HDL particles are comprised of several subfractions: HDL_2_, HDL_3_, apolipoprotein A-I (Apo A-I), apolipoprotein A-II (Apo A-II), and pre-*β*_1_. HDL_2_, Apo A-I, and pre-*β*_1_ are associated with cardioprotection and are found to be reduced in the obesity [35]. In response to the VLCD, Shoji et al. [29] observed that the initial decrease in HDL_2_ was associated with decreased lecithin cholesterol ester transfer protein (LCAT) (which plays an important role in the formation of HDL_2_ [36]) while Apo A-1 increased. Apo A-1 activates LCAT, which may explain the delayed improvement in HDL [29]. This observation is consistent with the meta-analysis by Dattilo and Kris-Etherton [37] that reported a decrease during active weight loss but an increase in HDL when weight loss was stabilised after a dietary intervention. More information, however, will be required to correlate weight, Apo-A1, and LCAT metabolism changes with HDL changes to allow a definitive comment on this parameter.

It has been suggested that exercise increases HDL cholesterol. In the studies reviewed here, Christiansen et al. displayed a significant increase in HDL. Fogelholm et al. [18] showed a similar trend, but this was not significant at 2 years. This was probably due to the fact that there was no difference in the number of steps taken between the different interventions at the end of the study, suggesting a poor compliance to exercise. Exercise has been associated with increases in HDL cholesterol, although a relatively high-intensity exercise is required for significant changes in HDL. It is thought that the increase in HDL in response to exercise is due to an increase in the activity of lipoprotein lipase (which transfers lipids to HDL) and a reduction in the activity of hepatic lipase (which removes lipids HDL in the blood) [38]. It has also been suggested that the degree of change in HDL in response to exercise may be dependent on genetic factors [39, 40]. 

The main concern about the use of VLCDs is the weight regain that is often observed after use. To date, there has not been any solution to the almost inevitable long-term weight regain associated with weight loss in general. While also focusing on the prevention of weight regain, however, it is important to also concentrate on maintaining the health benefits (i.e., increased HDL) associated with the initial weight loss. The study by Delbridge et al. [27] was quite interesting where they compared a high-carbohydrate to a high-protein weight-maintenance diet for a period of 12 months. Although there was a trend for an increase in HDL in both groups, there was no difference between the two weight-maintenance diets. This is inconsistent with the literature where it has been shown that at 12 months, HDL levels are generally higher in patients who followed a high-protein diet compared to a high-carbohydrate diet [41]. The effect of the weight maintenance diet in the study by Delbridge et al. [27] may have been blurred by the initial weight loss achieved using the VLCD, or by lack of compliance to the diet.

The effect of medication for weight maintenance on HDL could also be interesting to investigate. Four studies incorporated the use of obesity medication. One of the studies investigating the use of Orlistat showed no significant difference in HDL levels in the longer term [22] while the other study clumped their data for the placebo and Orlistat group for their overall analysis [24]. Erondu et al. [23] did not report a significant change in HDL at 1-year followup after randomisation to the neuropeptide-Y receptor agonist MK-0557. The study investigating the use of Sibutramine for weight maintenance, however, did report a long-term improvement in HDL. From other studies, it would appear that Sibutramine [25, 42] and dexfenfluramine [43] may be more effective for weight maintenance after a VLCD compared to Orlistat [15, 22, 44] or MK-0557 [23]. More research into the effect of Sibutramine on weight maintenance and the benefits on HDL should be pursued.

The main difficulty encountered was the heterogeneity of the studies reviewed here. There was a wide variety of intervention strategies, duration, and baseline HDL levels (ranging from 1.05 to 1.50 mmol/L) which makes it very difficult to reach clear conclusions and supply recommendations for best practice. In addition, there is a paucity of evidence for populations at increased risk of coronary artery disease such as type 2 diabetics, gender, and different ethnic groups. There is an obvious need for more research and better defined intervention strategies for the use of VLCDs in long-term treatment.

## 7. Conclusion

The results from this paper suggest that HDL can be improved in the longer term by using a combination of a VLCD followed by a weight-maintenance period using a hypocaloric diet. Nevertheless, the studies reviewed here are very heterogeneous and more research needs to be carried out to define the short-term effect of weight loss on HDL levels as well as investigating the effects of VLCDs in groups at higher risk of coronary heart disease such as type 2 diabetics.

## Figures and Tables

**Table 1:** Studies investigating the use of VLCD only.

Study	*N*	Number of males	Intervention	Duration of intervention	Weight at baseline (Kg)	Weight at end of intervention (Kg)	HDL at baseline (mmol/L)	HDL at end of intervention (mmol/L)
Clément et al. [13]	21	0	800 kcal/d	28 days	94 (3.0)	88 (3.0)*	1.05 (0.04)	0.9 (0.03)
Clément et al. [13]	8	0	650 kcal/d	2 days	120 (7.7)	119 (8)	1.4 (0.2)	—
Haugaard et al. [14]	13	4	600–800 kcal/d	8 weeks	106.4 (SE: 4.1)	97.0 (SE: 4.3)*	1.3 (SE: 0.1)	1.2 (SE: 0.1)*
Hong et al. [12]	152	0	Black participants 500–800 kcal/d + BT + exercise.	12 weeks	105 (21)	95 (28)*	1.45 (0.39)	1.34 (0.39)*
Hong et al. [12]	152	0	White participants 500–800 kcal/d + BT + exercise.	12 weeks	104 (28)	94 (25)*	1.50 (0.34)	1.37 (0.34)*
Laaksonen et al. [15]	20	9	800 kcal/d	9 weeks	101.3 (12.0)	86.4 (9.6)*	1.17 (0.26)	1.22 (0.18)
Lin et al. [16]	66	24	1200 kcal/d + 450 kcal/d	14 weeks (2 weeks LCD followed by 12 weeks VLCD)	92.5 (14.1)	∆ −8.37 (0.7)*	1.09 (0.20)	∆ −0.26 (0.85)
Lin et al. [16]	66	21	1200 kcal/d + 800 kcal/d	14 weeks (2 weeks LCD followed by 12 weeks VLCD)	92.1 (15.6)	∆ −8.42 (0.7)*	1.15 (0.25)	∆ −0.49 (0.85)

All values are reported as means (standard deviation) unless stated otherwise.

N: number of participants

*P < .05 compared to baseline

Δ Values reported as change

SE: standard error.

**Table 2:** Studies investigating the use of VLCDs followed by a maintenance phase.

Study	*N*	Number of males	Intervention	Duration of VLCD	Total duration of intervention	Weight at baseline (Kg)	Weight at end of VLCD(Kg)	Weight at end of study (Kg)	HDL at baseline (mmol/L)	HDL at end of VLCD (mmol/L)	HDL at end of study (mmol/L)
Apfelbaum et al. [25]	78	18	220–800 kcal/d followed by maintenance (hypocaloric balanced diet + placebo).	4 (± 1 week)	>12 month (4 ± 1 week of VLCD followed by 12 month double-blind treatment phase).	105.1 (20.3)	97.7 (19.7)*	Δ 0.5 (5.7)^†^	—	—	Δ 0.09 (0.08)
Apfelbaum et al. [25]	82	15	220–800 kcal/d followed by maintenance (hypocaloric balanced diet + sibutramine).	4 (± 1 week)	>12 month (4 ± 1 week of VLCD followed by 12 month double-blind treatment phase).	103.4 (17.5)	95.7 (16.9)*	Δ −5.2 (7.5)^∗†^	—	—	Δ 0.13 (0.09)*
Christiansen et al. [17]	29	15	600 kcal/d followed by maintenance (hypocaloric balanced diet).	8 week	12 weeks (8 weeks of VLCD followed by 4 weeks of maintenance).	107.8 (12)	Δ −11.2 kg*	95.5 (11)*	1.2 (0.3)	—	1.2 (0.3)
Christiansen et al. [17]	25	13	800 kcal/d + exercise followed by maintenance (hypocaloric balanced diet + exercise).	8 week	12 weeks (8 weeks of VLCD followed by 4 weeks of maintenance). Exercise was performed over the 12 weeks.	105.8 (15)	Δ −12.1 kg*	93.5 (13)*	1.2 (0.3)	—	1.3 (0.3)*
Delbridge et al. [27]	70	35	~500–550 kcal/d followed by maintenance (high-carbohydrate diet).	12 weeks	15 months (12 weeks VLCD; 12 months maintenance).	109.4 (2.6)	Δ −17.6 (0.8)*	Δ −14.3 (2.0)* (*n* = 40)	1.2 (0.04) (*n* = 65)	Δ −0.015 (0.02) (*n* = 62)	Δ − 0.21 (0.03)*(*n* = 33)
Delbridge et al. [27]	71	35	~500–550 kcal/d followed by maintenance (high-protein diet).	12 weeks	15 months (12 weeks VLCD; 12 months maintenance).	114.0 (3.0)	Δ −17.4 (0.7)*	Δ −14.8 (0.5)*(*n* = 42)	1.2 (0.03) (*n* = 59)	Δ 0.003 (0.03) (*n* = 54)	Δ 0.19 (0.04)* (*n* = 33)
Erondu et al. [23]	176	32	800 kcal/d followed by weight maintenance (hypocaloric diet + placebo).	6 weeks	58 weeks (6 weeks VLCD followed by 52 weeks maintenance).	101.5 (15.3)	92.3 (13.9)*	95.6 (15.7)*	1.45 (0.34)	1.26 (0.25)	1.48 (0.37)
Erondu et al. [23]	181	37	800 kcal/d followed by weight maintenance (hypocaloric diet + MK-0557).	6 weeks	58 weeks (6 weeks VLCD followed by 52 weeks maintenance).	98.4 (13.8)	89.5 (12.7)*	91.1 (14.5)*	1.41 (0.36)	1.22 (0.29)	1.48 (0.40)
Fogelholm et al. [18]	28	0	1 week LCD + 8 weeks VLCD + 3 weeks LCD. No increase in exercise.	8 weeks	12 weeks weight loss + 40 weeks no increased exercise + 2 year followup.	92.0 (9.8)	80.0 (9.5)*	89.7 (9.6) (*n* = 27)	1.22 (0.24)	1.12 (0.18)	1.34 (0.28) (*n* = 27)
Fogelholm et al. [18]	25	0	Same weight loss intervention as above + walking program targeted to expend 4.2 MJ/wk.	8 weeks	12 weeks weight loss + 40 weeks walking program (1) + 2 year followup.	92.0 (9.8)	78.0 (8.8)*	83.9 (12.2) (*n* = 24)	1.22 (0.24)	1.12 (0.27)	1.42 (0.35) (*n* = 24)
Fogelholm et al. [18]	27	0	Same weight loss intervention as above + walking program targeted to expend 8.4 MJ/wk.	8 weeks	12 weeks weight loss + 40 weeks walking program (2) + 2 year followup.	92.0 (9.8)	78.2 (11.6)*	87.4 (15.3) (*n* = 23)	1.22 (0.24)	1.13 (0.19)	1.41 (0.25) (*n* = 23)
Haugaard et al. [28]	9	2	600–800 kcal/d followed by weight maintenance (hypocaloric balanced diet).	8 weeks	32 weeks (8 weeks VLCD + 24 weeks weight maintenance).	104.5	94.7 (SE: 4.0)*	93.5 (SE: 4.9)*	1.4	1.2 (0.1)*	1.4 (0.1)
Heinonen et al. [24]	35	19	800 kcal/d followed by weight maintenance (hypocaloric balanced diet + placebo or Orlistat).	8 weeks	8 months (8 weeks VLCD + 6 months weight maintenance).	101.7 (2.0)	86.9 (1.7)*	86.6 (1.8)*	1.17 (0.04)	1.25 (0.04)*	1.23 (0.05)*
Paisey et al. [26]	15	6	Type 2 diabetic patients 450 kcal/d for women or 650 kcal/d for men.	6 weeks	>5 years (6 week VLCD and 5 year followup).	—	—	—	1.20 (0.39)	—	1.26 (0.47)
Pekkarinen et al. [20]	29	0	429 kcal/d followed by weight maintenance (refeeding to low-energy food). The programme included 12 sessions of behaviour methods for weight control.	8 week	52 week (8 week VLCD + 9 weeks refeeding and followup at week 52).	96.1 (10.9)	Δ −12.4 (3.3)*	Δ −10.7 (7.6)* (*n* = 27)	1.19 (0.25)	Δ −0.20 (0.20)*	Δ 0.16 (0.18)* (*n* = 26)
Richelsen et al. [22]	156	76	600–800 kcal/d followed by weight maintenance (hypocaloric balanced diet + Placebo).	8 weeks	38 months (8 weeks VLCD + 36 months placebo and hypocaloric diet).	111.9 (16.0)	Δ −14.3 *	Δ −7.2 *	1.15 (0.26)	Δ −0.07*	Δ 0.06*
Richelsen et al. [22]	153	76	600–800 kcal/d followed by weight maintenance (hypocaloric balanced diet + Orlistat).	8 weeks	38 months (8 weeks VLCD + 36 months Orlistat and hypocaloric diet).	110.7 (17.9)	Δ −14.5*	Δ −9.4*	1.13 (0.26)	Δ −0.05*	Δ 0.04*
Rolland et al. [19]	14	5	~550 kcal/d + behaviour therapy followed by weight maintenance (refeeding, balanced diet and behaviour therapy.	Average 6.9 months	9 months (12 weeks–9 months of VLCD followed by maintenance).	129.6 (23.0)	109.1 (14.6)*	109.1 (14.6)*	1.29 (0.19)	1.47 (0.23)*	1.47 (0.23)*
Wing et. [21]	17	4	LCD (1000 to 1500 kcal/d depending on initial body weight) followed by a VLCD (400 kcal/d) followed by weight maintenance (refeeding to hypocaloric diet) + BT (for duration of the study)	8 weeks	72 weeks (1 month LCD followed by 2 months of VLCD, followed by refeeding for 8 weeks and a followup after 52 weeks).	102.1 (11.7)	83.5 (9.5)*	93.5 (10.4)*	1.11 (2.3)	1.26 (0.30)*	1.33 (0.25)*

Values reported as means (standard deviation) unless stated otherwise.

N: number of participants

*P <.05 compared to baseline

SE: standard error

Δ values reported as change

^†^values do not include weight lost during the VLCD.

**Table 3:** Studies investigating the use of VLCDs as an intermittent treatment.

Study	*N*	Number of males	Intervention	Total duration of intervention	Weight at baseline (Kg)	Weight after 1st VLCD (Kg)	Weight at end of study (Kg)	HDL at baseline (mmol/L)	HDL after 1st VLCD (mmol/L)	HDL at end of study (mmol/L)
Lantz et al. [31]	117	35	450 kcal/d for 16 weeks, followed by a refeeding to a hypocaloric diet (minus 500 kcal/d). VLCD was repeated for 2 weeks every 3rd month or as soon as their body weight passed an individualised predetermined level.	>2 years (16 week VLCD followed by 3 week refeeding and 2 years of hypocaloric diet with intermittent or on demand VLCD)	113.9 (16.2)	Δ −14.9 (CI: −15.9; −13.9)*	Δ −9.6 (CI: −11.6; −7.7)*	1.2 (0.3)	Δ −0.1 (CI: −0.2; −0.07)*	Δ 0.2 (CI: 0.1; 0.2)*
Shoji et al. [8]	6	0	Intermittent LCD and VLCD. In the 1st LCD, energy intake was reduced from 1440 kcal to 1280 kcal to 880 kcal/d.In the 1st and 2nd VLCD periods, patients consumed 420 kcal/d. In the intermission period, the same 880 kcal meal used in the 1st LCD was given. In the 2nd LCD, daily energy intake was increased weekly from 880 kcal to 1280 kcal or 1440 kcal with the same menu used in the 1st LCD.	14-15 weeks (3 weeks LCD, 4 weeks VLCD, 1 week intermission, 4 weeks VLCD, 2-3 weeks LCD)	105 (range: 69–156)	Δ −12.7 (range: 9.6–16.0 range)	Δ −18.9 (range: 14.3 –31.4)*	1.10 (SE: 0.11)	0.81 (SE: 0.05)∗	0.95 (SE: 0.05)
Shoji et al. [29]	8	0	As described above	14–15 weeks (3 weeks LCD, 4 weeks VLCD, 1 week intermission, 4 weeks VLCD, 2-3 weeks LCD)	102 (range: 72–156)	—	Δ−18.5 Kg*	1.11 (0.07)	0.87 (0.06)*	0.98 (0.05)
Williams et al. [30]	18	9	Type 2 diabetic patients 1,500–1,800 kcal/d diet for 20 weeks except for a total of 20 study days during which they consumed 400–600 kcal/d VLCD. Patients followed the VLCD for 5 consecutive days during week 2 and then 1 day a week for 15 weeks.	20 weeks (LCD except for 20 days of intermittent VLCD)	103.5 (16.8)	—	Δ −9.6 (SE: 5.7)* (*n* = 16)	1.10 (0.20) (*n* = 16)	1.03 (017)*	1.13 (0.23)
Williams et al. [30]	18	7	Type 2 diabetic patients 1,500–1,800 kcal/d diet for 20 weeks except for a total of 20 study days during which they consumed 400–600 kcal/d VLCD.Patients followed the VLCD for 5 consecutive days during weeks 2, 7, 12, and 17.	20 weeks (LCD except for 20 days of intermittent VLCD)	104.8 (13.7)	—	Δ −10.4 (SE: 5.4)*(*n* = 15)	1.09 (0.17) (*n* = 15)	1.00 (0.15)*	1.08 (0.22)
Wing et al. [21]	45	15	400–500 kcal/d for weeks 1–12 and 24–36 of the programme. During the intermittent phases, refeeding to hypocaloric balanced diet (1000–1200kcal/d).	48 weeks (12 weeks VLCD, followed by 12 weeks refeeding, and then another 12 weeks VLCD, followed by 12 weeks refeeding)	105.8 (19.4)	Δ − 16.0 (5.8)*	Δ −14.2 (10.3)*	1.12 (0.21)	1.17 (0.23)*	1.25 (0.3)*

Values are reported as means (standard deviation) unless stated otherwise.

N: number of participants

*P < .05 compared to baseline

Δ Values reported as change

CI: confidence interval; SE: standard error.
